# Preparation of Cellulose Modified Wall Material Microcapsules and Its Effect on the Properties of Wood Paint Coating

**DOI:** 10.3390/polym14173534

**Published:** 2022-08-28

**Authors:** Yongxin Xia, Xiaoxing Yan, Wenwen Peng

**Affiliations:** 1 Co-Innovation Center of Efficient Processing and Utilization of Forest Resources, Nanjing Forestry University, Nanjing 210037, China; 2 College of Furnishings and Industrial Design, Nanjing Forestry University, Nanjing 210037, China

**Keywords:** cellulose, microcapsules, wood paint coating

## Abstract

An orthogonal experiment with four factors and three levels was designed. Nine different microcapsules were prepared by changing four factors: the core–wall ratio, emulsifier concentration, reaction temperature, and rotation speed. Through an analysis of the microcapsule yield and morphology, it was determined that the microcapsule of sample 6 performed the best in the orthogonal test and that the core–wall ratio was the largest factor affecting the microcapsule morphology and yield. In order to further optimize the performance of the microcapsules, single factor independent tests were carried out using the core–wall ratio as a single variable. It was found that the microcapsules with the core–wall ratio of 0.75:1 had good micro morphology and yield. The properties of the coating were the best when the microcapsules were added into the primer and the topcoat at the same time with an additional amount of 10.0%. The mechanical properties of the coating containing cellulose microcapsules and the coating without cellulose microcapsules were tested. Cellulose can enhance the toughness of the microcapsules, inhibit the generation of microcracks, and enhance the performance of the coating to a certain extent. The elongation at break of the coating with cellulose microcapsules was 9.49% higher than that without cellulose and was 11.1% higher than that without cellulose microcapsules.

## 1. Introduction

As a renewable and recyclable green material, wood plays an important role in production and in life [1,2,3,4]. Water-based paint film has inevitable defects in long-term use, as the insufficient toughness of the coating leads to unstable performance and micro cracks [5,6,7,8]. If the paint film is not repaired in time, the overall structure of the paint film is affected, a lot of human and material resources are consumed [9,10,11,12], and the cracks will be difficult to repair [13]. The generation of microcracks affects the integrity of paint film, thus reducing the service life of furniture, which is also an urgent problem to be solved at present [14,15,16].

As the cladding core material, the shell wall material should have a certain toughness and strength, and the core material should be released in time when cracks appear. The wall materials of microcapsules also need to have a stable structure and not react with the core materials, so that the core materials can be better stored and not allowed to flow out. Urea–formaldehyde resin is a common microcapsule wall material. The preparation method for urea–formaldehyde resin is relatively simple, but the urea–formaldehyde resin is brittle, which is not conducive to the preparation of paint film. Cellulose is a common, multifunctional, natural, and inexpensive material. It has good toughness and high mechanical strength and is widely used in various fields. Adding cellulose to the wall material of microcapsules can better improve the mechanical strength of the microcapsules and enhance the toughness of paint film.

The core material needs to be selected according to the use of the microcapsules. As a repair microcapsule added to water-based paint, the core material of the microcapsules needs to have good fluidity and curability [17]. The fluidity is mainly for the microcapsule to flow to the crack in time after the crack occurs on the surface of the paint film [18,19,20,21]. The core material should be harmless, natural, and green. Tung oil is a kind of pure, natural dry vegetable oil, which can be cured into a film by coming into contact with oxygen in the air at room temperature. It is an ideal core material [22,23,24].

In Peng et al. [17], microcapsules with different core–wall ratios were added to water-based coatings, and the influence of the core–wall ratio on the preparation of the microcapsules and the influence of the different contents of the microcapsules on the performance of the paint film were explored. In this paper, the effects of the core–wall ratio, emulsifier concentration, reaction temperature, and stirring rate on the properties of microcapsules were investigated using urea–formaldehyde and cellulose as composite wall materials and tung oil as the core material. Microcapsules containing cellulose were prepared using a four-factor and three-level orthogonal test, and the optimal factor affecting the preparation of microcapsules was determined. A good coating process for microcapsules containing cellulose being added to the paint film was explored. The performance of the coating containing cellulose microcapsules and the same coating without cellulose microcapsules was tested to explore the enhancement effect of the cellulose on the performance of the microcapsules and paint film.

## 2. Materials and Methods

### 2.1. Test Materials

The drugs and materials required for the preparation of microcapsules and paint films are shown in Table 1.

### 2.2. Preparation Method for Microcapsules

According to preliminary investigation [17], we found that a microcapsule with cellulose added into the wall material can be successfully prepared, and the preliminary investigation results show that the microcapsule with an added amount of cellulose around 1.0 g performed the best. Four factors and three levels of an orthogonal test were designed through changing the core–wall ratio, emulsifier concentration, rotation speed, and reaction temperature, and nine different microcapsules were prepared. The core–wall ratio refers to the mass ratio of the core material to the wall material that constitutes the microcapsule. By analyzing the appearance and yield of the microcapsules, a better microcapsule preparation process was determined. Then some microcapsules without cellulose in the wall material were prepared, and the performance of the microcapsules containing cellulose and of those without cellulose in the wall material were compared to explore the enhancement effect of cellulose on the performance of microcapsules. The preparation conditions for 9 kinds of test microcapsules are shown in Table 2, and the list of materials for microcapsule preparation is shown in Table 3.

The mass of the wall material was determined to be constant, and the core–wall ratio was changed by changing the mass of the core material. Firstly, the wall material was prepared. The 10.0 g urea and 13.5 g formaldehyde were completely reacted with a molar ratio of 1:1 to produce 15.0 g urea–formaldehyde resin. The urea and formaldehyde were mixed and stirred until the urea is fully dissolved, then the solution was put into a magnetic stirrer. The stirring temperature was adjusted to 70 °C, the stirring speed was adjusted to 600 r/min, and the stirring time lasted for 60 min. The triethanolamine was dropped in the above mixture to adjust the pH value of the solution to 8–9. The 1.0 g of cellulose was weighed and mixed with 50 mL water. After a reaction with urea–formaldehyde resin lasting 1 h, the cellulose solution was added and dispersed by ultrasound for 30 min.

The next step was the preparation of the core material. First, the emulsifier solution was prepared. Sodium dodecylbenzene sulfonate was used as the emulsifier, mixed with water and fully stirred, and then tung oil was added as the core material. The reaction temperature was adjusted to 45 °C, and the stirring speed was 1000 r/min. The tung oil was fully emulsified for 30 min. Then, the wall material mixed solution was added to the core material solution. After the solution was fully mixed, the monohydrate citric acid solution was added by the drop, the pH value was adjusted to 3–4, and 1–2 drops of n-octanol was added for defoaming to improve the yield, and the mixed solution was reacted for 2 h. After that, the mixed solution was allowed to stand for 5 days, and then the microcapsules of white powder were able to be obtained by the process of filtering, washing, and drying. Sample 10 is of the microcapsules without cellulose. The preparation process was the same as for the microcapsules described above, except that the wall material was urea–formaldehyde without cellulose.

### 2.3. Preparation Method for Coatings

In order to explore the influence of the addition of microcapsules in the primer and the topcoat on the performance of the waterborne paint film, three ways of adding microcapsules were set up. In the first, the microcapsules were only added to the primer, in the second, the microcapsules were only added to the topcoat, and in the third, the microcapsules were added to both the primer and the topcoat. The coating process adopted three bottoms and three sides, and the addition of microcapsules was fixed at 10.0%.

### 2.4. Testing and Characterization

A Quanta-200 scanning electron microscope (SEM) (Fei company of the United States, Hillsborough, OSU) and Zeiss sigma 300 optical microscope (OM) (Carl Zeiss company of Germany) were used to observe the micro morphology of the microcapsules and paint films. When using the scanning electron microscope, the sample was first adhered to the metal circular plate, and then the sample was plated with gold. Next, the sample was put into the instrument, the voltage of the SEM was set to 200 V–30 kV, the magnification to 20–300,000 times, and the resolution to 3.5 nm; then the microcapsules and paint film were observed. When an optical microscope was being used, first the sample was placed on the glass slide, then the glass slide was covered on the sample, and the sample was observed by adjusting the focal length and magnification.

The chemical composition of the microcapsules was measured using a VERTEX 80V infrared spectrometer (Brooke instruments, Germany). When measuring the powder, KBr was added first and was mixed with the sample and ground fully. Then, it was pressed into a transparent film by tablet pressing and put into the instrument for observation.

According to the national standard GB/T 6739-2006 [25] “Paints and Varnishes—Determination of Film Hardness by Pencil Test”, a QHQ pencil durometer (Shenzhen Forest Precision Instrument Co., Ltd., Shenzhen, China) was used to measure the hardness of the film. A pencil with a hardness of 6B-6H was placed on the mechanical trolley in turn. It was pressed down on the surface of the paint film at an angle of 45°, and then the trolley was driven evenly by force in order to leave a scratch on the surface of the paint film. If there was no scratch, the pencil was replaced with a harder pencil and the test continued until there was a 3 mm long scratch. The scratch results were observed with a magnifying glass; the greater the pencil H value, the stronger the hardness.

According to the national standard GB/T 4893.9-2013 [26] “Test of surface coatings of furniture—Part 9: Determination of resistance to impact”, the impact resistance of paint film was measured by a QCJ-120 impact tester (Shenzhen Suno Experimental Equipment Co., Ltd., Shenzhen, China). The side of the wood test plate coated with paint film was placed upward on the horizontal base of the impactor and fixe, then the impact ball was raised to a certain height, the switch was pressed, and the ball was allowed to fall freely to make impact with the plate. Marks and cracks were then observed at the impact position of the ball. If there were no impact marks and cracks, the falling height of the ball was increased and the process was repeated again until there were cracks in the paint film on the wood surface; the height of the small ball was recorded when the crack occurred.

Paint film was applied on the glass plate according to the coating process, and then the coating was taken off after the paint film was dry, and the paint film was removed from the glass plate. Then an MTest-i universal mechanical testing machine (Shanghai Yinti Precision Machinery Technology Co., Ltd., Shanghai, China) was used to test the elongation at break of the coating. The elongation was calculated at break of the paint film according to the calculation Formula (1), where L_0_ is the original length of the paint film, L is the length of the paint film at break, and e is the elongation at break of the paint film:

(1) $$e=\left[\left(L-L_{0}\right)÷L_{0}\right]\times 100\%$$

According to the national standard GB/T 4893.4-2013 [27] “Test of surface coatings of furniture—Part 4: Determination of adhesion—Cross cut”, a QFH scriber (Hebei Zhongke Beigong Test Instrument Co., Ltd., Cangzhou, China) was used to test the adhesion of the coating. A multi blade cutter was held and it was used to cut in the direction of approximately 45° with the wood surface, and the plate was rotated to cut it once at 90°, so as to finally cut it into a grid pattern. Then it was pasted on the grid with adhesive tape. In order to make full contact between the adhesive tape and the paint film on the wood surface, we used our fingernails to wipe the adhesive tape. Then the tape was torn off smoothly at an angle of about 60° within 0.5–1.0 s. The results were observed on the tape. The smaller the adhesion grade of the paint film, the less the paint film fell off and the smoother the cutting edge was. The classification table of adhesion grades is shown in Table 4.

Byes3200 precision roughness tester (Shanghai Bangyi Precision Meter Co., Ltd., Shanghai, China) was used to test the roughness of the paint film. The smoother the surface of the paint film, the smaller the R_a_ value.

According to GB/T 4893.6-2013 [28] “Test of surface coatings of furniture—Part 6: Determination of gloss value”, a LS195 intelligent gloss meter (Shenzhen Linshang Technology Co., Ltd., Shenzhen, China) was used to measure the gloss of the paint film at 20°, 60°, and 85°.

## 3. Results and Analysis

### 3.1. Analysis of Orthogonal Test Results of Microcapsules

#### 3.1.1. Yield Analysis of Microcapsules

The yield results of the microcapsules are shown in Table 5. Through the microcapsule production and range, the question of the biggest factor affecting the microcapsule production was explored. Through analysis, it could be found that the yield of microcapsules increased with the increase in core material content. The yields of sample 1, sample 6, and sample 8 were higher under the same core–wall ratio. Through the analysis of the range of microcapsules, the biggest factor affecting the yield of microcapsules was determined to be the core–wall ratio, while other factors and conditions in the preparation process had little influence on the microcapsules. Through our analysis of the mean value, we identified the best parameters for preparing microcapsules to be when the emulsifier concentration is 1.0%, the reaction temperature of the wall material and the wall material mixed solution is 30 °C, and the rotating speed is 900 r/min.

#### 3.1.2. Microanalysis of Microcapsules

**Figure 1:**
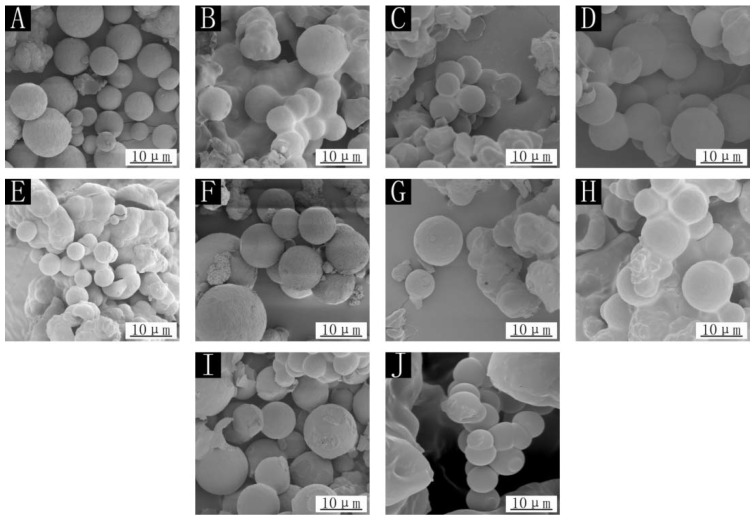
SEM of microcapsules: (**A**) sample 1, (**B**) sample 2, (**C**) sample 3, (**D**) sample 4, (**E**) sample 5, (**F**) sample 6, (**G**) sample 7, (**H**) sample 8, (**I**) sample 9, (**J**) sample 10.

The SEM images for nine kinds of microcapsules for the orthogonal test and sample 10 without cellulose are shown in Figure 1. By observing the scanning electron microscope, it was apparent that the prepared microcapsules were round; because the core material was liquid, the microcapsules were round. There was no obvious fine fiber outside the round spherical microcapsule, and the surface of the microcapsule was also relatively rough, so it could be inferred that the cellulose was in the wall material. Among them, the round microcapsules in samples 2, 3, 5, 7 and 8 were less shaped, and the agglomeration phenomenon was obvious. It can be judged that the microcapsules for these samples were also fewer. However, samples 1, 4, 6, and 9 had more microcapsules and less agglomeration of particles. Samples 1–9 with cellulose added to the wall material had a rough microcapsule surface in the figure, while the microcapsule surface of sample 10 was very smooth, mainly because cellulose was added to the wall material, which can make the surface of microcapsules rough. Based on the yield and morphology of the microcapsules, the best microcapsule preparation was obtained in sample 6.

**Figure 2:**
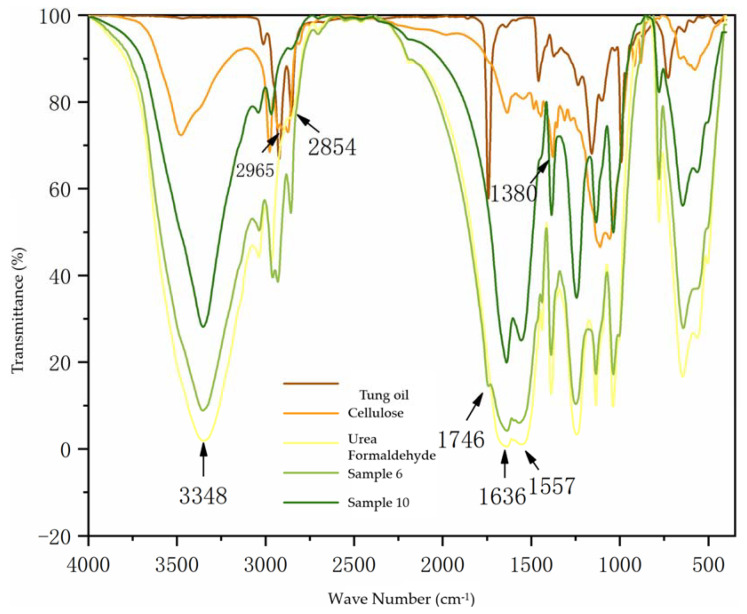
IR spectra of microcapsules, core material, and wall material.

Figure 2 shows the infrared spectrum analysis of the core material, wall material, and two kinds of microcapsules. It can be seen that 3348 cm^−1^ is the stretching vibration peak superimposed by N-H and O-H, 1636 cm^−1^ and 1557 cm^−1^ are the stretching vibration peaks of C=O and C-N, respectively. The absorption double peaks near 2965 cm^−1^ come from the stretching vibration of methylene in the molecular structure of cellulose, and the characteristic absorption peak at 1380 cm^−1^ is the stretching vibration of methyl groups, which are the characteristic peaks of wall urea-formaldehyde and cellulose [29,30]. This shows that the wall material of microcapsules exists. The stretching vibration peak of unsaturated bond C-H is 2854 cm^−1^, and 1746 cm^−1^ is the stretching vibration peak of C=O. These are the characteristic peaks of core tung oil, indicating that core tung oil also exists. The core and wall materials of the microcapsules can be found in the whole spectrum, which can prove that microcapsules contain core and wall materials. From the infrared spectra of the two microcapsules, it can be seen that the microcapsules of sample 10 do not have methylene double peaks from the molecular structure of cellulose near 2965 cm^−1^, indicating that there is no cellulose in the microcapsules, while other characteristic peaks are consistent with those of sample 6. Therefore, the microcapsules without cellulose were also successfully prepared.

### 3.2. Effect of Adding Methods on the Properties of Waterborne Paint Film

The changes in the gloss of the paint film are shown in Table 6. The three different light incidence angles of the paint film without microcapsules have high gloss, because the water-based paint film itself has good gloss. The gloss of the water-based paint film added with microcapsules decreased to a certain extent. Amongst the three addition methods, the gloss of the paint film with the microcapsules added in the topcoat decreased significantly. The reason is that the microcapsules were all added to the topcoat, resulting in the microcapsules being too concentrated on the surface. The content of the microcapsules added in the primer was too great and the microcapsules were too concentrated. However, after the primer had been brushed, the topcoat was brushed, so the gloss was better than it was when it was added in the topcoat. Adding the primer and topcoat at the same time made the microcapsules more evenly dispersed. By comparison, the gloss of the paint film prepared by adding the microcapsules into the primer and by adding the microcapsules into the primer and topcoat at the same time was better.

The hardness and adhesion of the water-based paint films using different addition methods were tested. The results for hardness and adhesion are shown in Table 7. The hardness of the coating with the microcapsules added in the primer and the coating with microcapsules added in the primer and topcoat at the same time was greater than that of the coating without microcapsules, indicating that adding microcapsules can enhance the hardness of the coating to a certain extent. The hardness of the coating with the microcapsules added to the topcoat was poor, mainly because the microcapsules were all mixed in the topcoat. Too much microcapsule content also meant that the microcapsules and paint film could not be mixed together well, thus affecting the interface compatibility between the water-based paint and microcapsules and consequently, the hardness of the paint film. Through the testing of the adhesion of the paint film, it was found that the adhesion level of the paint film with a microcapsule content of 10.0% increased, because the addition of microcapsules had a certain impact on the interfacial adhesion between the water-based paint and the wood, making the adhesion become worse. The paint film with microcapsules added to the topcoat was more likely to affect the adhesion, because the microcapsules were all concentrated in the topcoat. In general, the paint film prepared by adding the microcapsules into the primer and the paint film prepared by adding the microcapsules into the primer and topcoat at the same time both had better hardness and adhesion.

**Figure 3:**
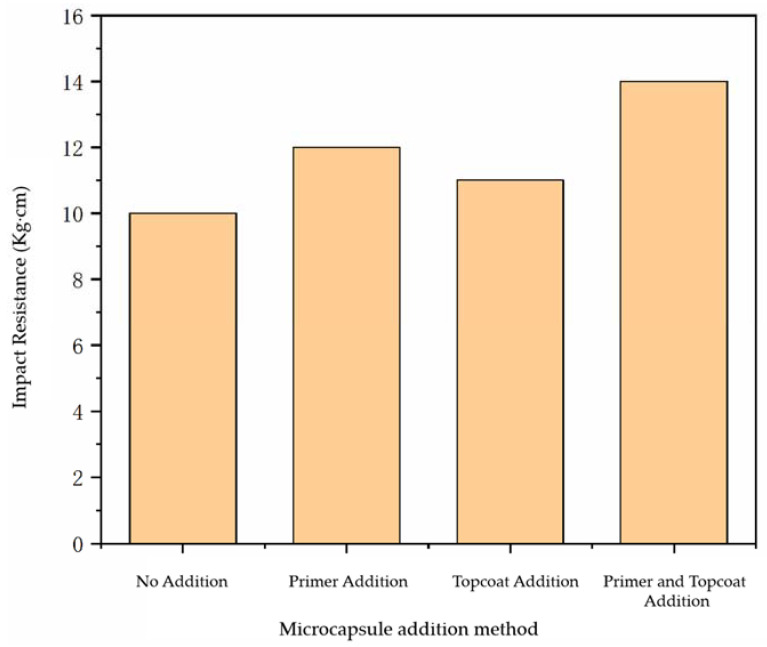
Influence of adding methods on the impact resistance of paint film.

The impact resistance of water-based paint film using different addition methods is shown in Figure 3. The impact resistance of the paint film prepared without microcapsules was 10.0 kg·cm, while the impact resistance of the paint film with microcapsules has been improved to a certain extent. The impact resistance of the paint film with the microcapsules added in the topcoat and the primer was 11.0 kg·cm and 12.0 kg·cm, respectively, while the impact resistance of the paint film with the microcapsules added in the primer and the topcoat at the same time was 14.0 kg·cm. The impact resistance of the paint film with microcapsules added in the primer and topcoat was not as strong as that of the paint film with the microcapsules added in the primer and topcoat at the same time, because although the microcapsules were able to improve the impact resistance of the paint film to a certain extent, the force between the paint film and the wood surface became diminished due to the agglomeration of microcapsules, so the paint film could not be well-attached to the wood surface. As a result, the impact resistance of the paint film was not so strong. It can be seen that the impact resistance of the paint film with the microcapsules added in the primer and topcoat at the same time was the best.

**Figure 4:**
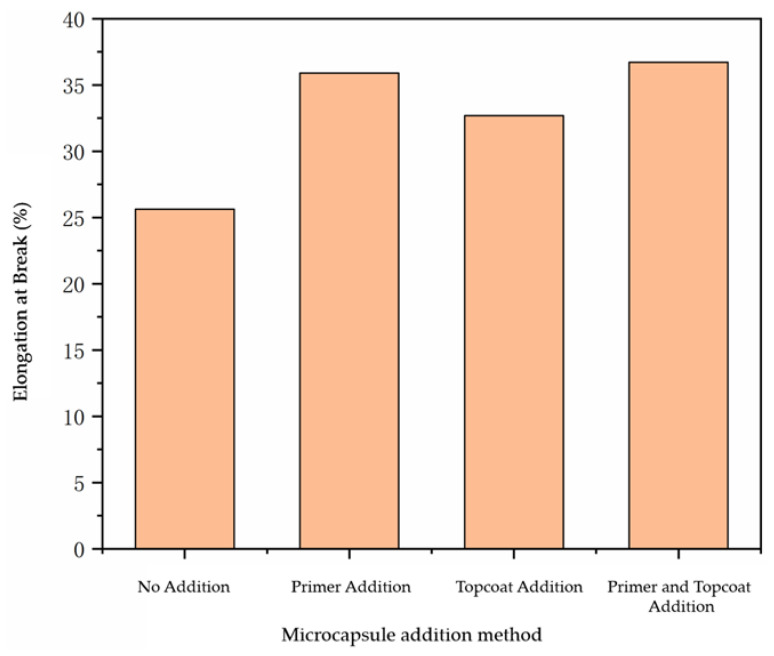
Influence of additive methods on the elongation at break of paint film.

The results for the elongation at break of the paint film prepared using the different addition methods are shown in Figure 4. From the figure, it can be seen that adding microcapsules can enhance the elongation at break of the paint film. The elongation at break of the paint film without microcapsules was only 25.62%. When the microcapsules with a content of 10.0% were added, the elongation at break of the paint film was able to be increased to 32.68–36.72%. Due to the different adding methods for the microcapsules, there were certain differences in the elongation at break of the paint film, but the differences were negligible. It can be concluded that the addition of microcapsules can improve the elongation at break of the paint film to a certain extent and improve the toughness of the paint film.

**Figure 5:**
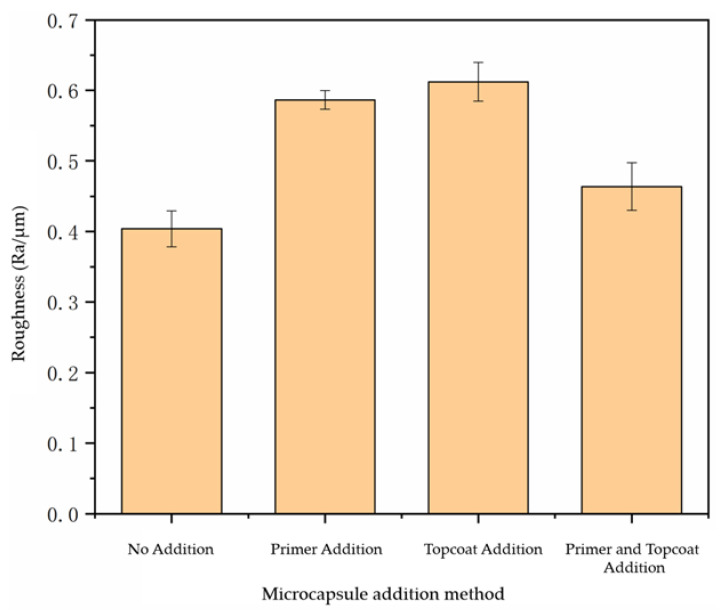
Influence of addition methods on the roughness of paint film.

By testing the roughness of the paint film prepared using the different adding methods, we can analyze whether the surface of the paint film was flat after adding microcapsules. In Figure 5, R_a_ represents the arithmetic average roughness, which was used to evaluate the overall roughness of the surface. It can be seen from the analysis that the roughness of the paint film without microcapsules was 0.42 μm. The roughness of the paint film with the microcapsules added in the primer and topcoat at the same time increased to 0.45 μm. The roughness of the paint film with the microcapsules added in the primer and topcoat increased to 0.59 μm and 0.61 μm, respectively. The surface of the paint film with the microcapsules added in the primer and topcoat at the same time became more uneven. This is because the microcapsules were too concentrated in the primer or topcoat, resulting in the agglomeration of microcapsules, which made the surface of the paint film rough. To sum up, the roughness of the paint film with microcapsules increased, and the paint film with the microcapsules added in the primer and topcoat at the same time had a smoother surface and less roughness than did the paint film with microcapsules added in the primer or topcoat only.

**Figure 6:**
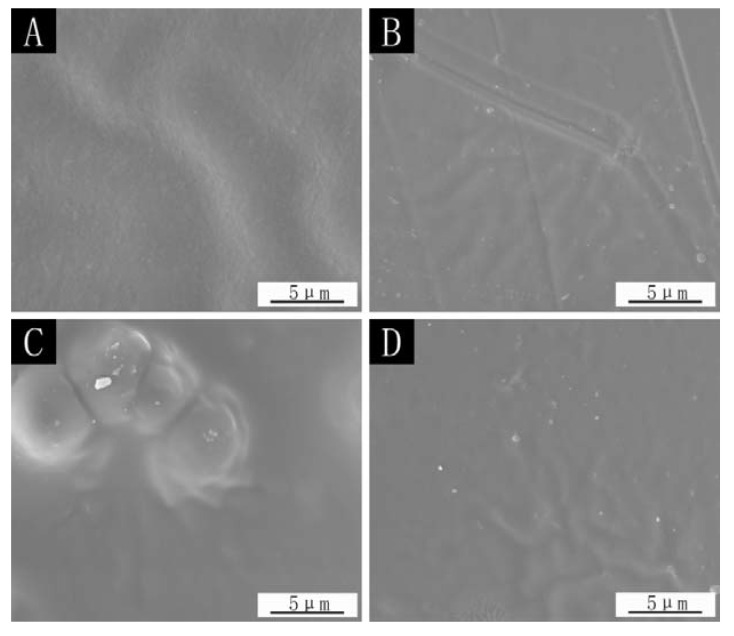
SEM images of the paint film surface in different addition methods: (**A**) the paint film without microcapsules, (**B**) microcapsules in primer, (**C**) microcapsules in topcoat, (**D**) microcapsules in primer and topcoat added at the same time.

Figure 6 is the SEM of the paint film surface using the different addition methods. From the figure, it can be seen that the paint film surface without microcapsules is smooth, and the ripples in the figure may be caused by the brushing process. There are particles on the surface of the paint film that are added with the microcapsules, mainly because the microcapsule itself is a kind of fine particle. The surface of the paint film with the microcapsules added in the topcoat shows an obvious swelling, which is caused by having many microcapsules in the topcoat and agglomeration. Finally, the microcapsules are agglomerated on the surface of the paint film, which affects the appearance and flatness of the paint film.

### 3.3. Effect of Cellulose Addition on Coating Properties

Through the research and analysis of microcapsule preparation, it is determined that the preparation process for sample 6 is the best one. The paint film without cellulose microcapsules was prepared through the process of coating three bottoms and three sides and the addition method of adding primer and topcoat paint at the same time was used. Then, the performance of the paint film without cellulose was tested to explore the influence of cellulose on the paint film.

Through the testing and analysis of the hardness, impact resistance, and elongation at break of the paint film prepared by sample 10 and compared with the paint film without microcapsules and with sample 6 microcapsules, the mechanical properties were determined and are shown in Table 8. It can be seen that adding microcapsules can enhance the performance of the paint film to a certain extent, but the hardness, impact resistance, and elongation at break of the paint film prepared by microcapsule sample 10 without cellulose were not significantly increased. Among the microcapsules with the same content, the microcapsules containing cellulose showed better performance than those without cellulose. The elongation at break of the paint film with sample 6 was 36.72%, which was 11.10% higher than for paint film without microcapsules. The elongation at break of the paint film with sample 10 was only 1.61% higher than that without microcapsules, mainly because the cellulose had strong mechanical properties, which strengthened the interfacial adhesion of the paint film surface and increased the toughness of the paint film. Because of these characteristics of cellulose, the hardness and impact resistance of the paint film containing cellulose were better than for those without cellulose.

## 4. Conclusions

Through the orthogonal test for four factors and three levels, the yield and microstructure of the microcapsules were analyzed, and the best factor affecting the preparation of the microcapsules was determined to be the core–wall ratio. By exploring the performance of the microcapsules with different addition methods, it was revealed that the gloss of the coating decreased to a certain extent. The hardness of the coating with the microcapsules was greater than that without microcapsules; the hardness of the coating with primer and a topcoat added at the same time could reach 5H. The adhesion of the coating with the microcapsules was generally decreased, and the adhesion of the coating with the microcapsules added in primer and a topcoat at the same time was better; the adhesion grade was grade 1. The impact resistance and elongation at break of the coating were the best when the microcapsules were added into the primer and the topcoat at the same time. The impact resistance was 14.0 kg·cm and the elongation at break reached 36.72%. The roughness of the coating was the least when the microcapsules were added in the primer and the topcoat at the same time. The hardness, impact resistance, and elongation at break of the coating with the microcapsules containing cellulose in the wall material were better than in the coating without cellulose. Cellulose was able to increase the toughness of the coating to a certain extent, inhibit the generation of microcracks, and improve the performance of the coating.

## Figures and Tables

**Table 1:** List of chemicals and materials used in the test.

Material Name	Specification	Place of Origin
Cellulose	Analytical purity	Hebei Jinzhong Cellulose Technology Co., Ltd., Jinzhou, China
37 wt.% Formaldehyde solution	Analytical purity	Jiangsu Changchun Chemical Co., Ltd., Changshu, China
Urea	Analytical purity	Nanjing Panfeng Chemical Co., Ltd., Nanjing, China
N-octanol	Analytical purity	Jiangsu Anyi Chemical Co., Ltd., Hai’an, China
Citric acid monohydrate	Analytical purity	Shandong Lemon Biochemical Co., Ltd., Anqiu, China
Triethanolamine	Analytical purity	Suqian Yongsheng Fine Chemical Company, Suqian, China
Sodium dodecyl benzene sulfonate	Analytical purity	Tianjin Beichen Fangzheng Reagent Factory, Tianjin, China
Tung oil	—	Guangzhou Chaoya Chemical Co., Ltd., Guangzhou, China
Dulux waterborne wood primer	—	Dulux Co., Ltd., Shanghai, China
Dulux waterborne wood topcoat	—	Dulux Co., Ltd., Shanghai, China
Board	50 mm × 100 mm × 8 mm	Beijing Tiantan Furniture Co., Ltd., Beijing, China

**Table 2:** Orthogonal experiment on the preparation parameters for microcapsules.

Sample	Core–Wall Ratio	Emulsifier Concentration (%)	Temperature (°C)	Rotating Speed (r/min)
1	0.50:1	1.0	30	600
2	0.50:1	2.0	50	900
3	0.50:1	3.0	70	1200
4	0.75:1	1.0	30	1200
5	0.75:1	2.0	50	600
6	0.75:1	3.0	70	900
7	1:1	1.0	30	900
8	1:1	2.0	50	1200
9	1:1	3.0	70	600

**Table 3:** List of materials prepared for the microcapsule test.

Sample	Urea (g)	Formaldehyde Solution (g)	Cellulose (g)	Tung Oil (g)	Sodium Dodecyl Benzene Sulfonate (g)	Deionized Water (mL)
1	10.0	13.5	1.0	1.0	0.8	79.2
2	10.0	13.5	1.0	1.0	0.8	39.2
3	10.0	13.5	1.0	1.0	0.8	25.8
4	10.0	13.5	1.0	1.0	1.2	118.8
5	10.0	13.5	1.0	1.0	1.2	58.8
6	10.0	13.5	1.0	1.0	1.2	38.8
7	10.0	13.5	1.0	1.0	1.6	158.4
8	10.0	13.5	1.0	1.0	1.6	78.4
9	10.0	13.5	1.0	1.0	1.6	51.7

**Table 4:** Paint film adhesion grade classification sheet.

Adhesion (Grade)	Illustrate
1	The edges and corners of the grid do not fall off the paint film
2	The edge of the grid has obvious paint film peeling
3	The edges and corners of the grid have obvious paint film peeling and the peeling area is greater than 15% and less than 35%
4	The edges and corners of the grid have obvious paint film peeling and the peeling area is greater than 35% and less than 65%

**Table 5:** Microcapsule yield and range results.

Sample	Core–Wall Ratio	Emulsifier Concentration (%)	Temperature (°C)	Rotating Speed (r/min)	Yield (g)
1	0.50:1	1.0	30	600	9.23
2	0.50:1	2.0	50	900	9.05
3	0.50:1	3.0	70	1200	9.11
4	0.75:1	1.0	30	1200	11.43
5	0.75:1	2.0	50	600	11.40
6	0.75:1	3.0	70	900	12.15
7	1:1	1.0	30	900	12.67
8	1:1	2.0	50	1200	12.87
9	1:1	3.0	70	600	11.26
Mean value 1	9.130	11.110	11.417	10.630	
Mean value 2	11.660	11.107	10.580	11.290	
Mean value 3	12.267	10.840	11.060	11.137	
Range	3.137	0.270	0.837	0.660	

**Table 6:** Influence of adding methods on the gloss of the paint film.

Microcapsule Addition Method	Gloss (%)		
	20°	60°	85°
No addition	52.9 ± 1.1	78.5 ± 1.1	89.6 ± 1.6
Primer addition	28.7 ± 2.6	45.8 ± 2.2	53.1 ± 2.5
Topcoat addition	12.5 ± 0.6	18.0 ± 1.1	21.6 ± 1.7
Primer and topcoat addition	25.5 ± 2.7	57.6 ± 3.7	54.8 ± 1.9

**Table 7:** Effect of adding methods on hardness and adhesion of paint film.

Microcapsule Addition Method	Hardness	Adhesion (Level)
No addition	3H	0
Primer addition	4H	1
Topcoat addition	3H	2
Primer and topcoat addition	5H	1

**Table 8:** Mechanical properties of paint films prepared by microcapsules of different wall materials.

Paint Films	Hardness	Impact Resistance (kg·cm)	Elongation at Break (%)
Paint film without microcapsules	3H	10.0	25.62
Paint film with the microcapsules of sample 6	5H	14.0	36.72
Paint film with the microcapsules of sample 10	4H	11.0	27.23

## Data Availability

Not applicable.
